# High-Risk VREfm Clones and Resistance Determinants in a Thai Hospital

**DOI:** 10.3390/antibiotics14030229

**Published:** 2025-02-24

**Authors:** Peechanika Chopjitt, Rada Kansaen, Sumontha Chaisaeng, Sawarod Phongchaiwasin, Parichart Boueroy, Piroon Jenjaroenpun, Thidathip Wongsurawat, Anusak Kerdsin, Nuchsupha Sunthamala

**Affiliations:** 1 Faculty of Public Health, Kasetsart University Chalermphrakiat, Sakon Nakhon Campus, Sakon Nakhon 47000, Thailand; peechanika.c@ku.th (P.C.); rada.kan@ku.th (R.K.); sumontha.chai@ku.th (S.C.); parichart.bou@ku.th (P.B.); anusak.ke@ku.th (A.K.); 2 Department of Biology, Faculty of Science, Mahasarakham University, Maha Sarakham 44150, Thailand; am.sawarod@gmail.com; 3 Division of Medical Bioinformatics, Research Department, Faculty of Medicine Siriraj Hospital, Mahidol University, Bangkok 10700, Thailand; piroonj@gmail.com (P.J.); thidathip@gmail.com (T.W.)

**Keywords:** antimicrobial resistance, bacteremia, *Enterococcus faecium*, sequence typing, vancomycin, whole-genome sequencing

## Abstract

**Background/Objective:** Vancomycin-resistant enterococci (VRE), particularly *Enterococcus faecium* (VREfm), are significant healthcare-associated infections, especially bloodstream infections (BSIs). **Method:** This study explored the genotypic and phenotypic characteristics of 29 VREfm isolates causing BSIs in Thailand. Bacterial species, sequence types (STs), virulence genes, and vancomycin antimicrobial-resistance genes were identified by multiplex PCR, multilocus sequence typing, and whole-genome sequencing (WGS). Antibiotic susceptibility was determined by disk diffusion, while an E-test or broth microdilution were used for daptomycin, teicoplanin, linezolid, and tigecycline. Biofilm formation was assessed using a microtiter plate assay. **Results:** All isolates harbored the *vanA* gene and exhibited resistance to ampicillin, erythromycin, norfloxacin, vancomycin, and rifampin. Resistance to ciprofloxacin, tigecycline, and nitrofurantoin was widespread as well. All isolates remained susceptible to chloramphenicol and linezolid. The majority of isolates belonged to clonal complex 17, with ST17 being predominant (21/29, 72.4%), followed by ST80 (6/29, 20.7%), ST761 (1/29, 3.4%), and ST117 (1/29, 3.4%). WGS analysis confirmed the presence of various antimicrobial resistance genes, including *aac*(*6*′)*-Ii*, *ant-Ia*, *erm*(*B*), and *vanA*. Additionally, virulence genes such as *acm* (collagen adhesin) and *esp* (enterococcal surface protein), which are involved in biofilm formation, were detected. **Conclusion:** This study provides insights into the genomic characteristics and clonal dissemination of invasive VREfm in Thailand, which is crucial for infection control and public health surveillance.

## 1. Introduction

*Enterococcus* species are Gram-positive bacteria inhabiting diverse environments, including the gastrointestinal tracts of humans and animals, as well as soil and water. These opportunistic microbes have emerged as significant pathogens, causing various community-acquired and healthcare-associated infections such as urinary tract infections, endocarditis, intravenous catheter-related infections, surgical site infections, bacteremia, and neonatal sepsis [1,2,3].

Among enterococci, *Enterococcus faecium* (*E. faecium*) and *Enterococcus faecalis* (*E. faecalis*) are the most clinically relevant, ranking as the second and third most prevalent nosocomial pathogens globally [4,5,6]. The increasing antimicrobial resistance among these species, particularly the greater propensity for multidrug resistance in *E. faecium* compared to *E. faecalis*, presents a critical global health challenge [7]. The World Health Organization has classified vancomycin-resistant *E. faecium* (VREfm) as a “high-priority pathogen”, highlighting the urgent need for new antibiotics [8]. Hospital-associated *E. faecium* lineages, particularly clonal complex 17, pose significant healthcare challenges due to their virulence and vancomycin resistance [9]. The predominance of CC17 in hospital settings necessitates rapid typing methods to facilitate targeted infection control measures and mitigate the substantial burden of these infections [10].

*E. faecium* has emerged as a major cause of healthcare-associated bloodstream infections worldwide [11,12], and vancomycin resistance significantly complicates treatment. Various studies have identified *Enterococcus* spp. as the fourth most common pathogen in bloodstream infections, while a systematic review of community-acquired BSIs in South and Southeast Asia revealed *Enterococcus* spp. were the third most prevalent Gram-positive bacteria [13]. Epidemiological data from Europe indicate that the proportion of vancomycin-resistant *E. faecium* (VREfm) in bloodstream isolates increased from 8.1% in 2012 to 19.0% in 2018, with notable regional variations. For instance, in 2018, VREfm proportions were 28.4% in Northern Europe and 32.0% in Eastern Europe, while Western and Southern Europe reported lower proportions of 11.2% and 15.3%, respectively [14]. Mortality associated with *E. faecium* BSIs has been reported at 30% and 35% [15,16]. Careful monitoring and molecular characterization of vancomycin-resistant *E. faecium* is crucial to understanding the local epidemiology, identify high-risk clonal lineages, and inform the development of targeted prevention and control strategies [17]. 

Resistance to vancomycin may be intrinsic or acquired, with nine distinct vancomycin resistance gene clusters identified to date: *vanA*, *vanB*, *vanC*, *vanD*, *vanE*, *vanG*, *vanL*, *vanM*, *vanN*, and *vanP*. However, only the *vanA* and *vanB* clusters are prevalent, which are typically acquired through horizontal gene transfer, likely due to their association with successful mobile genetic elements (MGEs) [18,19]. 

While numerous studies have investigated the molecular characteristics of resistance, virulence, and clonality in local VRE strains [20,21,22,23], including our previous study on urinary isolates [24], there is a significant gap in understanding the molecular profiles of VREfm from bloodstream infections in Thailand. Bloodstream infections are associated with higher morbidity and mortality rates compared to urinary tract infections, underscoring the need for focused research in this area. In our previous study, we identified 4 STs among 16 urinary VREfm isolates, with ST17 being the most prevalent. However, molecular characteristics of bloodstream isolates remain largely unexplored. This study aimed to characterize the resistance profile and sequence types of 29 VREfm strains isolated from bloodstream infections in Thai tertiary hospitals, thereby enhancing our understanding of their molecular epidemiology and informing targeted treatment and infection control strategies.

## 2. Results

### 2.1. Antimicrobial Resistance Profile

Antimicrobial susceptibility testing of the VREfm isolates revealed that all were classified as multidrug-resistant (MDR) bacteria, exhibiting 100% resistance to ampicillin, erythromycin, norfloxacin, rifampin, and vancomycin. Furthermore, a majority of the VREfm strains were resistant to ciprofloxacin (96.6%), tigecycline (89.7%), and nitrofurantoin (86.2%). Conversely, all VREfm isolates showed susceptibility to chloramphenicol and linezolid (Table 1).

### 2.2. Detection of Vancomycin Resistance Genes

**Figure 1:**
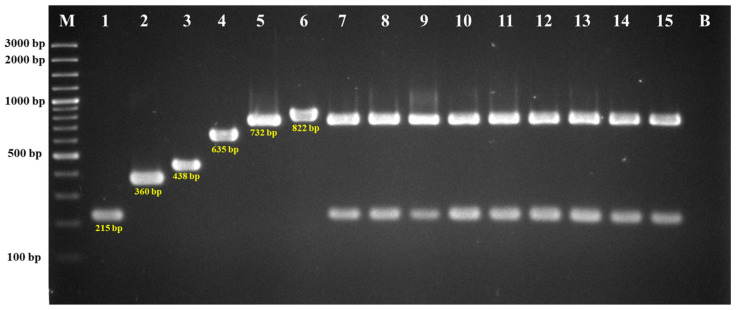
Multiplex PCR detection of vancomycin-resistant *Enterococcus faecium*. Lane M is a 100 bp DNA marker, lane 1 is *E. faecium* (215 bp), lane 2 is *E. faecalis* (360 bp), lane 3 is *vanC2/C3* (438 bp), lane 4 is *vanB* (635 bp), lane 5 is *vanA* (732 bp), lane 6 is *vanC1* (822 bp), lane 7–15 are positive samples, and lane B is the negative control.

All strains carried the *vanA* gene, confirming a glycopeptide-resistant phenotype (Figure 1). None of the other vancomycin resistance genes (*vanB*, *vanC1-C3*) were detected.

### 2.3. Biofilm Formation

Of the 29 VREfm strains, biofilm-forming capacity production ranged from 1.2 to 2.1. Two isolates were identified as moderate biofilm producers, biofilm-forming capacity 2.0–2.1, demonstrating a greater capacity to form substantial biofilm structures compared to the majority of the strains. These moderate biofilm producers harbored virulence genes such as *esp* (enterococcal surface protein) and *acm* (collagen adhesin), which are known to enhance biofilm formation by promoting cell-to-cell adhesion and attachment to surfaces. The remaining 27 VREfm strains were classified as weak biofilm producers (biofilm-forming capacity = 1.2–1.8), demonstrating a limited ability to form biofilms. These weak biofilm producers lacked or expressed lower levels of *esp* and *acm*, suggesting a direct role of these virulence factors in biofilm production (Table 1).

### 2.4. Multi Locus Sequence Typing (MLST)

Genetic analysis by MLST, which characterizes VREfm using the sequences of internal fragments of seven housekeeping genes (*atp*, *ddl*, *ghd*, *purK*, *gyd*, *pstS*, and *adk*), revealed seven different sequence types (STs) among the isolates. The most prevalent sequence type was ST17 (n = 21), followed by ST80 (n = 6), ST117 (n = 1), and ST761 (n = 1). The results of the MLST analysis are presented in Table 1.

### 2.5. Whole-Genome Sequencing

The whole-genome sequencing data revealed a total of 18 distinct virulence genes within the genomes of five representative VREfm strains. The *aac*(*6*′)*-Ii*, *ant*(*6*)*-Ia*, *msr*(*C*), *erm*(*B*), *acm*, *espfm*, and *vanA* genes were found in all strains. Notably, two virulence factors were identified: *acm* (collagen adhesin) and *espfm* (enterococcal surface protein), which contribute to biofilm formation and were found in all VREfm samples. The five VREfm strains subjected to whole-genome sequencing included one strain that exhibited moderate biofilm formation, consistent with the WGS results revealing the presence of the *espfm* gene.

Plasmid analysis of genomic sequences from the five VREfm strains using PlasmidFinder revealed the presence of nine distinct plasmid replicon sequences. Notably, rep2, rep11a, and repUS15 were present in all isolates. The *rep2* replicon has been predominantly associated with human isolates and is linked to the presence of the *vanA* gene, conferring vancomycin resistance. Similarly, rep11a and repUS15 have been identified in plasmids carrying *vanA*, suggesting their role in antimicrobial resistance dissemination. These findings indicate that the identified plasmids may contribute to the virulence and resistance profiles of the VREfm isolates. Additionally, rep17, rep12, repUS43, rep14a, and repUS7 were also detected in the strains. Furthermore, the *vanHAX* genes were located on the rep17 replicon type in four strains, and another was located on the rep1/rep2 plasmid replicon types, as shown in Table 2.

### 2.6. Phylogenetic Analysis

**Figure 2:**
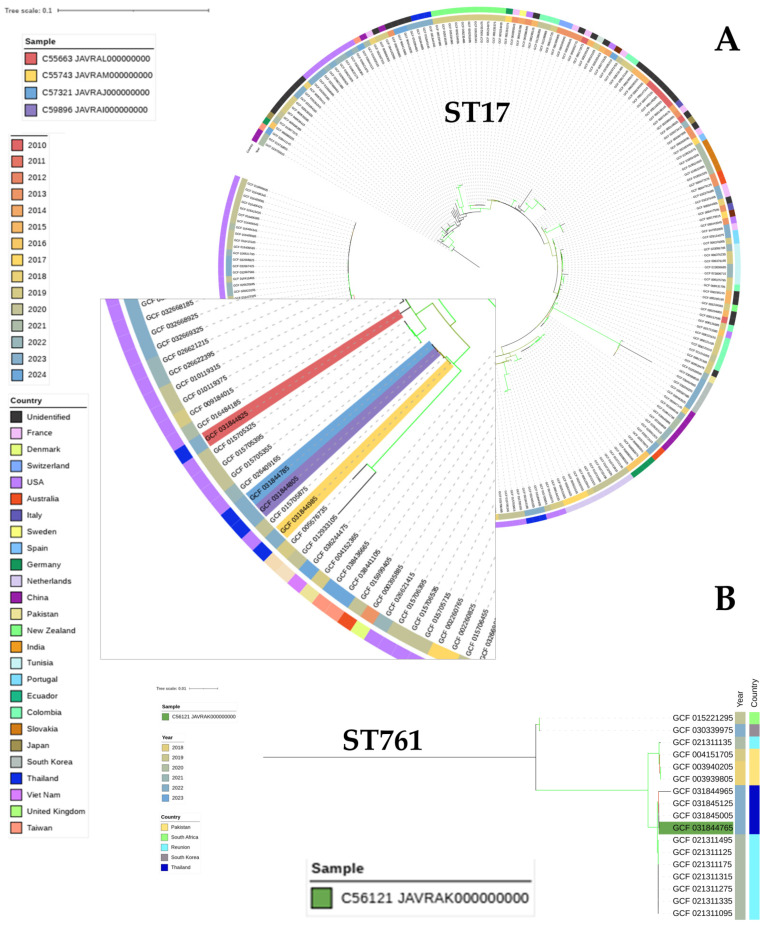
The whole-genome core-genome phylogenetic tree of the five VREfm strains generated by Snippy and visualized using the interactive tree of life tool. (**A**) The analysis reveals four of VREfm strains in our studies belong to ST17. (**B**) One strain of VREfm belongs to ST761.

Phylogenetic analysis based on sequence types revealed that isolates C55663, C57321, C59896, and C55743 were divided into two distinct clusters. The first cluster includes C55663, which is closely related to VREfm strains isolated from clinical specimens in the United States in 2020 (GCA_015705325.1) and 2021 (GCA_016484185.1). The second cluster comprises C57321, C59896, and C55743, showing close genetic relatedness to strains from the USA in 2020 (GCA_015705875.1) and 2022 (GCA_026409165.1), as well as India in 2019 (GCA_005576735.1). Additionally, C56121 belongs to ST761 and is closely related to strains from Thailand in 2023 (GCA_031845005.1) and Reunion in 2021 (GCA_021311495.1) (Figure 2). The presence of these clusters indicates potential international dissemination of specific VREfm lineages. The close genetic relatedness between our isolates and those from the USA, India, Thailand, and Reunion suggests possible global transmission routes, which could be facilitated by international travel, trade, or healthcare-associated transfers. The identification of ST761 in both Thailand and Reunion highlights the potential for regional dissemination within Asia and neighboring regions. These findings emphasize the need for global surveillance and collaborative efforts to monitor and control the spread of VREfm.

## 3. Discussion

This study provides insights into the molecular epidemiology and virulence characteristics of bloodstream-invasive VREfm in Thailand. All invasive VREfm isolates had the *vanA* gene, which makes them resistant to vancomycin and other glycopeptide antibiotics. Antimicrobial resistance patterns of our invasive VREfm were similar to previous reports in Thailand [25] and other countries [26,27,28,29]. The presence of the *vanA* genes, responsible for high-level vancomycin resistance, is particularly concerning as it compromises the effectiveness of this last-resort antibiotic.

All 29 VREfm strains were completely resistant to ampicillin, erythromycin, norfloxacin, rifampin, and vancomycin. Consistent with other studies, VREfm isolates have shown high resistance to multiple antibiotic classes, including commonly used antimicrobials [17,25,30,31,32,33,34,35,36]. This multidrug-resistance profile highlights the significant challenge these strains pose in clinical settings, where treatment options are increasingly limited. These findings emphasize the significant antibiotic resistance profile of the VREfm strains isolated in this study. 

The capacity of VREfm to form biofilms is a significant virulence factor that contributes to its persistence within hospital settings and increased tolerance to antimicrobials. Our investigation revealed that the majority of VREfm isolates exhibited weak biofilm-forming ability, with only two strains demonstrating moderate biofilm production. Previous studies have identified numerous VREfm strains capable of producing biofilms [37,38]. The varying biofilm-forming abilities of different VREfm isolates may stem from the complexity of biofilm formation, which depends on numerous genetic and environmental factors. The lower biofilm formation in this study may be due to variations in experimental conditions or strain characteristics. The degree of biofilm formation in VREfm has notable clinical implications. Even weak biofilm-forming strains can adhere to surfaces and medical devices, facilitating colonization and persistence in hospital environments. A study by Goudarzi et al. (2018) found that 49% of VREfm isolates were capable of biofilm production, highlighting their potential to persist in clinical settings [39]. In contrast, moderate biofilm-forming strains are more likely to cause chronic infections and treatment failures due to their enhanced structural integrity and increased tolerance to antibiotics. Previous studies have demonstrated that *E. faecium* isolates with higher biofilm-forming capacity exhibit increased resistance profiles, complicating treatment strategies [40,41]. Biofilms formed by VREfm have been shown to reduce the penetration and efficacy of antibiotics, leading to prolonged infections and higher morbidity rates. A recent study by Li et al. (2023) evaluated the antimicrobial and antibiofilm activity of essential fatty acids against VREfm, underscoring the challenges posed by biofilm-associated antibiotic resistance [42]. Additionally, biofilms facilitate the exchange of genetic material, including antibiotic resistance genes, which can accelerate the spread of resistance within bacterial populations. Consistent with these findings, Arndt et al. reported that 55% of VREfm isolates showed increased biofilm production, which may contribute to enhanced desiccation tolerance and genetic exchange [43].

MLST analysis revealed seven distinct STs among the VREfm isolates, indicating genetic diversity. The most prevalent ST was ST17, followed by ST80, ST117, and ST761. These STs belong to the global CC 17 lineage, which is often associated with heightened antibiotic resistance and the presence of virulence factors such as *esp* and *hyl.* These characteristics are often linked to outbreaks in healthcare settings [44]. Notably, several studies have established ST17 as the most widespread ST globally [45,46,47], and its prevalence among Thai VREfm isolates from blood samples aligns with previous findings that identify this ST as a predictor of bacteremia [44,48]. Epidemiological evidence consistently highlights ST17 and ST80 as two of the most frequently encountered and clinically significant VREfm lineages worldwide [36,49,50,51,52]. All VREfm isolates analyzed in this study were classified as high-risk CC, including CC17. These bacteria are of particularly concern due to their increased antibiotics resistance, greater transmissibility, and heightened likelihood of causing nosocomial outbreaks [36,49,53,54]. The dominance of CC17 among the VREfm isolates in Thailand is likely attributable to its superior fitness, enhanced colonization ability, and robust resistance mechanisms, which provide adaptive advantages in healthcare settings. The detection of these high-risk STs underscores the widespread dissemination of globally circulating VREfm clones and their establishment within Thai healthcare settings.

Genomic analysis revealed the presence of *aac*(*6*′)*-Ii*, *aac*(*6*′)*-aph*(*2*″), *aph*(*2*′)*-la* conferring resistance to gentamicin, *ant*(*6*′)*-la* mediating resistance to streptomycin, *msr*(*C*) and *erm*(*B*) resistance to erythromycin, *tet*(*L*), *tet*(*M*) resistance to tetracycline, only one strain of VREfm found *dfrG* mediating resistance to trimethoprim according to previous studies [55,56]. Furthermore, additional resistance determinants, such as *Inu*(*B*) resistance to lacosamide, and *vanA* resistance to vancomycin and teicoplanin, were also detected. These additional determinants correspond to and explain the phenotypic resistance profiles observed in the isolates, confirming the genotypic–phenotypic correlation. The detection of virulence genes, such as *acm* and *esp*, suggests the potential for enhanced colonization, biofilm formation, and persistence within the host [55]. While our study assessed biofilm formation in vitro, further investigation is needed to understand the role of these virulence factors in clinical outcomes.

VRE can spread in two main ways: vertical spread via clonal dissemination, and horizontal transfer via mobile genetic elements such as plasmids [26]. The plasmid analysis revealed a diversity of plasmid replicons, some of which carried the *vanA* genes. Among these, rep17 was identified as the most common plasmid type associated with *vanA* genes in the VREfm isolates, consistent with previous studies [30,57,58,59]. Additionally, one strain harbored *vanA* genes on rep1/rep2 plasmid replicon types, which have also been previously linked to vancomycin resistance genes [30,57,58,60]. The presence of *vanA*-carrying plasmids across multiple replicon types highlights the potential for horizontal gene transfer of vancomycin resistance among enterococcal isolates within hospital environments and patient populations. This underscores the adaptability of these resistance mechanisms and their ability to spread across different genetic backgrounds. The diversity of plasmid replicons carrying *vanA* genes has significant implications for infection control strategies in healthcare settings [61,62]. The mobilization of resistance genes across various plasmid types facilitates the rapid dissemination and persistence of vancomycin-resistant enterococci (VRE), complicating efforts to contain outbreaks [63]. This genetic flexibility suggests that resistance can spread not only through clonal expansion but also via plasmid-mediated horizontal transfer, even among genetically distinct strains [62,64]. Consequently, infection control measures must extend beyond traditional approaches, such as patient isolation and antibiotic stewardship, to include enhanced surveillance of plasmid-mediated resistance mechanisms [61]. Environmental monitoring and stricter disinfection protocols are also critical, as plasmids can persist on surfaces and medical equipment, serving as reservoirs for resistance gene transfer [63]. Furthermore, the identification of specific plasmid replicons, such as rep17 and rep1/rep2, could inform targeted molecular surveillance programs to track and mitigate the spread of high-risk resistance determinants [61]. By addressing the role of plasmid diversity in resistance dissemination, healthcare facilities can develop more effective, multifaceted strategies to combat the spread of VRE and other multidrug-resistant pathogens [62].

The phylogenetic analysis revealed that the VREfm isolates were genetically similar to strains previously reported from the United States and India, suggesting the potential for intercontinental dissemination and global spread of these high-risk clonal lineages [50]. This finding highlights the transnational nature of the VREfm epidemic and the need for coordinated global surveillance and infection control efforts to limit the further spread of these clinically significant and difficult-to-treat multidrug-resistant strains. The close genetic relatedness among isolates from geographically distant regions underscores the remarkable ability of these VREfm clones to traverse international borders, potentially through human travel, patient transfer between healthcare facilities, or other transmission routes. Tracing the global epidemiology of these high-risk VREfm lineages is crucial for understanding their dispersal patterns and informing targeted interventions to curb their continued dissemination within and across national boundaries.

In conclusion, we demonstrated that the invasive *vanA*-harboring VREfm isolates are multidrug-resistant and largely belong to a global high-risk clone (CC17). All five representative invasive VREfm contained a plasmid harboring *vanA*, suggesting horizontal transfer potential. However, these invasive VREfm are still susceptible to linezolid and chloramphenicol.

## 4. Materials and Methods

### 4.1. Bacterial Identification

A total of 29 VREfm strains were isolated from blood samples collected from patients in Thailand. The strains were cultured on blood agar (HiMedia Laboratories Pvt. Ltd., Nashik, India) and incubated for 18 h at 37 °C. Initial colonies were identified using Gram staining and standard biochemical tests to confirm their characteristic enterococcal morphology [65]. The species identity of all isolates was then confirmed through species-specific multiplex polymerase chain reaction (PCR), as described by Jackson et al. [66]. Finally, all isolates were stored at −80 °C for future analysis.

### 4.2. Antimicrobial Susceptibility Testing

Antimicrobial susceptibility testing for the VREfm isolates was performed using the disk diffusion method on Mueller–Hinton agar (bioMerieux, Marcy-l’Étoile, France), following the guidelines of the Clinical and Laboratory Standards Institute (CLSI 2024) [67]. Briefly, bacterial suspensions equivalent to a 0.5 McFarland standard were swabbed onto Mueller–Hinton agar plates. Antibiotics-impregnated disks were placed on the inoculated plates, which were then incubated at 37 °C for 18–24 h. The zones of inhibition diameters were measured, and the results were interpreted according to CLSI (2024) criteria. The following antibiotics were tested: ampicillin (AM, 10 μg), erythromycin (E, 15 μg), tetracycline (TE, 30 μg), ciprofloxacin (CIP, 5 μg), norfloxacin (NX, 10 μg), nitrofurantoin (FM, 300 μg), chloramphenicol (C, 30 μg), fosfomycin (FOS, 200 μg), vancomycin (VA, 30 μg), and rifampin (RA, 5 μg). The minimum inhibitory concentrations (MICs) of daptomycin (DAP), teicoplanin (TEC), and linezolid (LZD) were determined using an E-test (Liofilchem S.r.l., Roseto degli Abruzzi, Italy), with incubation at 35°C for 18 h. The MIC of tigecycline was determined by broth microdilution following the protocols established by the European Committee on Antimicrobial Susceptibility Testing (EUCAST, 2024), using an incubation time at 35 °C for 18 h [68]. *E. faecium* ATCC 29212 and *S. aureus* ATCC 25923 were used as quality controls.

### 4.3. Multiplex Polymerase Chain Reaction

DNA extraction from the isolates was performed using the GF-1 Bacterial DNA Extraction Kit (Vivantis, Selangor Darul Ehsan, Malaysia). Bacterial colonies were suspended in 20 μL of lysis buffer, and incubated at 95 °C for 20 min. Subsequently, 180 μL sterile deionized water (DI) was added to the mixture, which was then stored at −20 °C until further use. Species-specific markers and vancomycin resistance genes *vanA*, *vanB*, and *vanC* were identified using multiplex PCR [66,69]. The PCR reaction was prepared in a final volume of 25 μL, containing 12.5 μL of 2X JumpStart™ REDTaq^®^ ReadyMix™ Reaction Mix (Sigma-Aldrich Co. LLC., St. Louis, MO, USA), 0.5 μM of each forward and reverse primer, and 2 μL of DNA sample, and DI water to adjust the volume. The amplification protocol consisted of an initial denaturation step at 95 °C for 4 min, followed by 30 cycles of denaturation (95 °C, 30 s), annealing (55 °C, 1 min), and extension (72 °C, 1 min), with a final extension step (72 °C, 7 min). The PCR products were analyzed by gel electrophoresis, stained with ethidium bromide, and visualized under UV transillumination (SynGene; Cambridge, UK). A DNA molecular weight marker (DNA Ladder Thermo Scientific; Vilnius, Lithuania) was used as a standard, and *Enterococcus faecium* ATCC 19434 served as a positive control.

### 4.4. Biofilm Formation Assay

The ability of the VREfm isolates to form biofilms was determined using the microtiter plates assay, as previously described [70]. Briefly, bacterial isolates were first grown in 3 mL of trypticase soy broth (TSB, HiMedia Laboratories Pvt. Ltd., Nashik, India) with 1% glucose, then incubated at 37 °C under aerobic conditions for 24 h. The cultures were then adjusted to a turbidity equivalent to 0.5 McFarland using TSB. Next, 100 µL of this suspension, containing approximately 10^6^ CFU, was added to each well of a 96-well microplate prefilled with TSB. The plate was then incubated at 37 °C for both 24 and 48 h. After incubation, the wells were gently emptied of their contents and rinsed twice with deionized water. Once air-dried, 200 µL of 0.1% crystal violet was added to stain the adherent biofilm for 10 min. The wells were then washed twice with deionized water, air-dried again, and treated with 200 µL of absolute ethanol, which was allowed to sit at room temperature (25 °C) for 5 min. To measure absorbance, 100 µL of the ethanol-staining solution was transferred to a new 96-well plate and read at 595 nm using a spectrophotometer. Each strain was assessed in triplicate across three separate experimental runs. Absorbance values were averaged and standard deviations calculated. The mean absorbance readings were normalized against a negative control. Biofilm production was classified as follows: non-adherent (absorbance below 1), weak (1–2), moderate (2–4), or strong (above 4).

### 4.5. Multilocus Sequence Typing (MLST)

Multilocus sequence typing (MLST) was carried out as described previously [71], focusing on seven housekeeping genes (*atpA*, *ddl*, *gdh*, *purK*, *gyd*, *pstS*, and *adk*). These genes were amplified by PCR and sequenced. Each PCR reaction mixture (50 μL) contained 12.5 μL 2× MytaqTM HS Red Mix (Bioline Reagents Ltd.; London, UK), 2.5 μM of forward and reverse primers, 100 ng of bacterial DNA sample, and sterile deionized water. The thermal cycling protocol consisted of an initial denaturation at 94 °C for 3 min, followed by 35 cycles of denaturation at 94 °C for 30 s, annealing at 50 °C for 30 s, and extension at 72 °C for 30 s, and the cycle was completed with a single extension at 72 °C for 5 min. The PCR amplicons were purified using a GF-1 AmbiClean Kit (Vivantis Technologies Sdn Bhd; Kuala Lumpur, Malaysia) and sent for sequencing at 1st BASE products and services company, Malaysia. The resulting sequences were analyzed to determine alleles and sequence types (STs) using the scheme published in multilocus sequence typing databases https://pubmlst.org/organisms/enterococcus-faecium, accessed on 7 July 2024.

### 4.6. Whole-Genome Sequencing and Bioinformatic Analysis

Whole-genome sequencing was performed using an MGISEQ-2000RS (MGI Tech Co., Ltd., Shenzhen, China) and MiniON (Oxford Nanopore Technologies; Oxford, UK) platforms to analyze representatives of the VREfm isolates (n = 5). These isolates were selected based on their antimicrobial profile, geographic location, and virulence factors. This selection aimed to capture a diverse range of genetic backgrounds and resistance mechanisms, which is crucial for understanding the epidemiology and transmission dynamics of VREfm in the studied population. Bacterial DNA was extracted using a ZymoBIOMIC DNA miniprep Kit (Zymo Research; Irvine, CA, USA) according to the manufacture’s procedure. DNA concentration and purity were assessed with a Nanopore 2000 spectrophotometer (Thermo Scientific, Wilmington, DE, USA). Genomic DNA was digested using restriction enzymes, ligated to an adaptor, and the final PCR-amplified library fragments were evaluated. DNA libraries were prepared using the MGIEasy FS DNA Library Prep Set (MGI Tech Co., Ltd., Shenzhen, China). The libraries were quantified using a Qubit 2.0 Fluorometer (Invitrogen, Carlsbad, CA, USA) and sequenced using an MGISEQ-2000RS platform (MGI Tech Co., Ltd., Shenzhen, China) with a 150 bp paired-end.

For ONT sequencing, the rapid barcoding DNA sequencing protocol was used with the SQK-RBK004 kit (Oxford Nanopore Technologies; Oxford, UK), and the libraries were sequenced on a MinION Mk1B sequencer (Oxford Nanopore Technologies; Oxford, UK) with a single R10 version/FLO-MIN106D flow cell. The raw data were base-called and demultiplexed using the Guppy v5.0.11 (ONT) program, and the ONT adapters were trimmed using the Porechop v0.2.4 program2 [72]. Quality control of the ONT reads was performed with NanoPlot v1.28.1 program [73]. Hybrid assemblies combining the ONT and MGISEQ-2000 data were generated using Unicycler v0.5.0 [74] and the genome sequences were checked for quality using the QUAST v5.0.2 program [75]. The genome sequences were annotated using the DDBJ Fast Annotation and Submission tool [76]. Default parameters were used for all software programs unless otherwise stated. The ST was identified using the MLST 2.0 program [77]. Antibiotic resistance genes were detected based on the Comprehensive Antibiotic Resistance Database [78] and ResFinder4.1 software [79]. Plasmid replicon and virulence genes were analyzed using PlasmidFinder2.1 [80], and VirulenceFinder2.0 [81,82] programs.

Following core genome analysis of *E. faecium*, a total of the 3780 *E. faecium* genomes and their corresponding metadata (e.g., assembly accession, year, location) were retrieved from the NCBI database on 18 June 2024. Among these, five genomes, including GCF_031844825.1 (C55663 JAVRAL000000000), GCF_031844985.1 (C55743 JAVRAM000000000), GCF_031844765.1 (C56121 JAVRAK000000000), GCF_031844785.1 (C57321 JAVRAJ000000000), and GCF_031844805.1 (C59896 JAVRAI000000000), represent Thai samples. The genomes were classified based on seven housekeeping genes (*atpA*, *ddl*, *gdh*, *purK*, *gyd*, *pstS*, and *adk*) employing MLST [83]. Core genome multi-locus sequence typing (cgMLST) was conducted using 1423 cgST loci downloaded from Ridom on 21 June 2024. The resulting cgMLST profiles were used to construct a minimum spanning tree (MST) with Grapetree [84]. Genomes that belonged to the same ST as the Thai sample were analyzed for core single nucleotide polymorphism (core-SNP) using Snippy [85]. Alignment of core-SNPs sequences facilitated construction of a core-genome phylogenetic tree using the BioNJ distance method with 100 bootstraps. The resulting tree was visualized using iTOL [86], annotating with year, location, and STs.

### 4.7. Data Availability

The assembled genomic sequences were deposited in the NCBI Genbank Database under the Bioproject accession number PRJNA1002621.

## 5. Conclusions

This study provides comprehensive insights into the molecular epidemiology and resistance characteristics of VREfm isolates from a healthcare setting in Thailand. The identification of high-risk sequence types, such as ST17, along with the detection of a diverse array of antibiotic resistance and virulence genes, highlights the significant clinical and public health implications of these isolates. The dissemination of multidrug-resistant and highly virulent VREfm strains, driven by the mobilization of resistance genes on diverse plasmid backgrounds, poses a substantial threat to patient safety and healthcare systems. Ongoing surveillance, effective infection control measures, and the development of new treatment strategies are essential to mitigate the spread and impact of these dangerous pathogens.

## Figures and Tables

**Table 1:** Antimicrobial susceptibility profiles of 29 VERfm strains.

ID	ST	Resistance Profile	Biofilm Formation	Disk Diffusion Assay										Minimum Inhibitory Concentrations			
				AM	E	TE	CIP	NX	FM	C	RA	FOS	VA	DAP	TEC	**LZD**	**TG**
C21999	117	AM-E-CIP-NX-FM-RA-VA-TG	moderate	R	R	S	R	R	R	S	R	S	R	SDD	S	S	R
C54282	17	AM-E-CIP-NX-FM-RA-VA-TG	weak	R	R	S	R	R	R	S	R	S	R	SDD	I	S	R
C54284	80	AM-E-TE-NX-RA-FOS-VA-DAP-TG	weak	R	R	R	S	R	S	S	R	R	R	R	I	S	S
C55013	17	AM-E-CIP-NX-FM-RA-VA-TG	weak	R	R	S	R	R	R	S	R	S	R	S	I	S	R
C55449	17	AM-E-CIP-NX-FM-RA-VA-TG	weak	R	R	S	R	R	R	S	R	S	R	SDD	I	S	R
C55643	17	AM-E-TE-CIP-NX-RA-VA-TG	weak	R	R	S	R	R	S	S	R	S	R	SDD	S	S	R
C55663	17	AM-E-TE-CIP-NX-FM-RA-VA-DAP-TEC-TG	weak	R	R	R	R	R	R	S	R	S	R	R	R	S	R
C55743	17	AM-E-TE-CIP-NX-FM-RA-VA-TG	weak	R	R	R	R	R	R	S	R	S	R	SDD	S	S	R
C56086	80	AM-E-CIP-NX-RA-Fos-VA-TEC-TG	weak	R	R	S	R	R	S	S	R	R	R	S	R	S	R
C56087	17	AM-E-CIP-NX-FM-RA-DAP-VA-TG	weak	R	R	S	R	R	R	S	R	S	R	R	I	S	R
C56121	761	AM-E-TE-CIP-NX-FM-RA-VA-TEC-TG	moderate	R	R	R	R	R	R	S	R	I	R	SDD	R	S	R
C56263	17	AM-E-TE-CIP-NX-FM-RA-VA-DAP-TG	weak	R	R	R	R	R	R	S	R	S	R	R	I	S	R
C56321	17	AM-E-TE-CIP-NX-FM-RA-VA-TG	weak	R	R	S	R	R	R	S	R	S	R	SDD	I	S	R
C56881	80	AM-E-TE-CIP-NX-FM-RA-VA	weak	R	R	R	R	R	R	S	R	S	R	SDD	I	S	S
C56885	17	AM-E-TE-CIP-NX-FM-RA-VA-TEC-TG	weak	R	R	R	R	R	R	S	R	S	R	S	R	S	R
C56960	80	AM-E-TE-CIP-NX-FM-RA-VA-TEC-TG	weak	R	R	R	R	R	R	S	R	I	R	S	R	S	R
C57006	17	AM-E-CIP-NX-FM-RA-VA-TG	weak	R	R	S	R	R	R	S	R	I	R	S	S	S	R
C57321	17	AM-E-TE-CIP-NX-FM-RA-VA-TEC-TG	weak	R	R	R	R	R	R	S	R	S	R	S	R	S	R
C57552	17	AM-E-CIP-NX-FM-RA-VA-TG	weak	R	R	S	R	R	R	S	R	S	R	SDD	I	S	R
C57553	17	AM-E-CIP-NX-FM-RA-VA-TG	weak	R	R	S	R	R	R	S	R	S	R	SDD	I	S	R
C57891	17	AM-E-CIP-NX-FM-RA-VA-TEC-TG	weak	R	R	S	R	R	R	S	R	S	R	SDD	R	S	R
C58032	17	AM-E-TE-CIP-NX-FM-RA-VA-DAP-TEC-TG	weak	R	R	R	R	R	R	S	R	S	R	R	R	S	R
C58893	17	AM-E-CIP-NX-FM-RA-VA-DAP-TEC-TG	weak	R	R	S	R	R	R	S	R	S	R	R	R	S	R
C58898	17	AM-E-TE-CIP-NX-FM-RA-VA-DAP-TEC-TG	weak	R	R	R	R	R	R	S	R	S	R	R	R	S	R
C58999	80	AM-E-CIP-NX-RA-VA	weak	R	R	S	R	R	S	S	R	R	R	SDD	S	S	S
C59614	17	AM-E-TE-CIP-NX-FM-RA-VA-TG	weak	R	R	R	R	R	R	S	R	S	R	SDD	I	S	R
C59896	17	AM-E-TE-CIP-NX-FM-RA-VA-TEC-TG	weak	R	R	R	R	R	R	S	R	S	R	SDD	R	I	R
C973058	80	AM-E-TE-CIP-NX-FM-RA-VA-TG	weak	R	R	R	R	R	R	S	R	S	R	S	I	S	R
C980087	17	AM-E-TE-CIP-NX-FM-RA-VA-DAP-TG	weak	R	R	R	R	R	R	S	R	S	R	R	S	S	R

ST; sequence type; AM; Ampicillin, E; Erythromycin, TE; Tetracyclin, CIP; Ciprofloxacin, NX; Norfloxacin, FM; Nitrofurantoin, C; Chloramphenicol, RA; Rifampin, FOS; Fosfomycin, VA; Vancomycin, DAP; Daptomycin, TEC; Teicoplanin, LZD; Linezolid, TG; Tigecycline; R; resistance, I; intermediate, S; susceptible, SDD; susceptible-dose-dependent.

**Table 2:** Genomic features *of* five representative vancomycin-resistant *Enterococus feacium*.

Isolate ID	Accession No.	Total Length (bp)	No. of Contigs	GC Content (%)	N50 Value (bp)	Location	Size (bp)	AMR Gene	Virulence Genes
C55663	JAVRAL000000000	3,208,776	7	37.8%	2,868,836	Chromosome (repUS43)	2,868,836	*aac*(*6*′)*-li*, *tet*(*L*), *tet*(*M*), *msrC*	*acm*, *espfm*
						Plasmid1 (repUS15)	236,386	*aac*(*6*′)*-aph*(*2*″)	
						Plasmid2 (rep2)	45,304	*ant*(*6*′)*-la*, *aph*(*3*′)*-lll*, *erm*(*B*), *tet*(*L*), *Inu* (*B*)	
						Plasmid3 (rep17)	41,309	*ant*(*6*′)*-la*, *aph*(*3*′)*-lll*, *erm*(*B*), *VanHAX*	
						Plasmid4	6303	-	-
C55743	JAVRAM000000000	3,186,481	11	37.6%	2,796,351	Chromosome	2,796,351		*acm*, *espfm*
						Plasmid1 (repUS15)	231,840	*aac*(*6*′)*-aph*(*2*″), *aph*(*2*″)*-la*	
						Plasmid2 (repUS7)	66,682		
						Plasmid3 (rep17)	41,112	*ant*(*6*′)*-la*, *aph*(*3*′)*-lll*, *erm*(*B*), *VanHAX*	
						Plasmid4 (repUS12)	24,090	*aph*(*3*′)*-llla*, *erm*(*B*), *aph*(*3*′)*-lll*	
						Plasmid5	6303		
						Plasmid6 (rep11a)	6173		
C56121	JAVRAK000000000	3,286,524	12	37.6%	2,862,459	Chromosome (repUS12, (repUS43)	2,862,459	*erm*(*T*), *dfrG*, *aac*(*6*′)*-Ii*, *tet*(*L*), *msr*(*C*)	*acm*, *espfm*
						Plasmid1 (repUS15)	286,985	*aac*(*6*′)*-aph*(*2*″), *aph*(*2*″)*-la*	
						Plasmid2 (rep17)	41,941	*ant*(*6*)*’la*, *erm*(*B*), *aph*(*3*′)*-III*, *VanHAX*	
						Plasmid3 (rep2)	31,183	*ant*(*6*)*-la*, *erm*(*B*), *aph*(*3*′)*-III*, *Inu*(*B*)	
						Plasmid4	6303		
						Plasmid5 (rep11a)	6173		
						Plasmid6	5202		
C57321	SAMN37176629	3,208,752	16	37.8%	2,916,952	Chromosome	2,916,952		*acm*, *espfm*
						Plasmid1 (repUS15)	181,040		
						Plasmid2 (rep17)	41,655	*ant*(*6*′)*-la*, *aph*(*3*′)*-lll*, *erm*(*B*), *VanHAX*	
						Plasmid3 (rep2)	39,977	*aph*(*3*′)*-III*, *ant*(*6*)*-la*, *erm*(*B*), *tet*(*M*), *tet*(*L*), *Inu*(*B*)	
						Plasmid4 (rep11a)	6303		
						Plasmid5	6173		
C59896	JAVRAI000000000	3,250,015	9	37.7%	2,893,187	Chromosome	2,893,187		*acm*, *espfm*
						Plasmid1 (repUS15)	253,837	*aac*(*6*′)*-aph*(*2*″), *aph*(*2*″)*-la*	
						Plasmid2 (rep1, rep2)	43,995	*ant*(*6*′)*-la*, *erm*(*B*), *VanHAX*	
						Plasmid3	34,023		
						Plasmid4	8059		
						Plasmid5	6303		

## Data Availability

Data are contained within the article.

## References

[1] Fisher K., Phillips C. (2009). The ecology, epidemiology and virulence of *Enterococcus*. Microbiology.

[2] Kibwana U.O., Manyahi J., Moyo S.J., Blomberg B., Roberts A.P., Langeland N., Mshana S.E. (2024). Antimicrobial resistance profile of *Enterococcus* species and molecular characterization of Vancomycin resistant *Enterococcus faecium* from the fecal samples of newly diagnosed adult HIV patients in Dar es Salaam, Tanzania. Front. Trop. Dis..

[3] Amuasi G.R., Dsani E., Owusu-Nyantakyi C., Owusu F.A., Mohktar Q., Nilsson P., Adu B., Hendriksen R.S., Egyir B. (2023). *Enterococcus* species: Insights into antimicrobial resistance and whole-genome features of isolates recovered from livestock and raw meat in Ghana. Front. Microbiol..

[4] Lee T., Pang S., Abraham S., Coombs G.W. (2019). Antimicrobial-resistant CC17 *Enterococcus faecium*: The past, the present and the future. J. Glob. Antimicrob. Resist..

[5] Guzman Prieto A.M., van Schaik W., Rogers M.R.C., Coque T.M., Baquero F., Corander J., Willems R.J.L. (2016). Global emergence and dissemination of *enterococci* as nosocomial pathogens: Attack of the clones?. Front. Microbiol..

[6] Bender J.K., Cattoir V., Hegstad K., Sadowy E., Coque T.M., Westh H., Hammerum A.M., Schaffer K., Burns K., Murchan S. (2018). Update on prevalence and mechanisms of resistance to linezolid, tigecycline and daptomycin in *enterococci* in Europe: Towards a common nomenclature. Drug Resist. Updates.

[7] Werner G., Coque T.M., Hammerum A.M., Hope R., Hryniewicz W., Johnson A., Klare I., Kristinsson K.G., Leclercq R., Lester C.H. (2008). Emergence and spread of vancomycin resistance among *enterococci* in Europe. Euro Surveillance: Bull. Eur. Sur Les Mal. Transm. = Eur. Commun. Dis. Bull..

[8] Sadowy E., Luczkiewicz A. (2014). Drug-resistant and hospital-associated *Enterococcus faecium* from wastewater, riverine estuary and anthropogenically impacted marine catchment basin. BMC Microbiol..

[9] Permana B., Harris P.N.A., Runnegar N., Lindsay M., Henderson B.C., Playford E.G., Paterson D.L., Beatson S.A., Forde B.M. (2023). Using Genomics to Investigate an Outbreak of Vancomycin-Resistant *Enterococcus faecium* ST78 at a Large Tertiary Hospital in Queensland. Microbiol. Spectr..

[10] Top J., Willems R., Bonten M. (2008). Emergence of CC17 *Enterococcus faecium*: From commensal to hospital-adapted pathogen. FEMS Immunol. Med. Microbiol..

[11] Mahony A.A., Buultjens A.H., Ballard S.A., Grabsch E.A., Xie S., Seemann T., Stuart R.L., Kotsanas D., Cheng A., Heffernan H. (2018). Vancomycin-resistant *Enterococcus faecium* sequence type 796—*Rapid* international dissemination of a new epidemic clone. Antimicrob. Resist. Infect. Control.

[12] Tedim A.P., Ruíz-Garbajosa P., Rodríguez M.C., Rodríguez-Baños M., Lanza V.F., Derdoy L., Zurita G.C., Loza E., Cantón R., Baquero F. (2017). Long-term clonal dynamics of *Enterococcus faecium* strains causing bloodstream infections (1995–2015) in Spain. J. Antimicrob. Chemother..

[13] Zhang Y., Du M., Chang Y., Chen L.A., Zhang Q. (2017). Incidence, clinical characteristics, and outcomes of nosocomial *Enterococcus* spp. bloodstream infections in a tertiary-care hospital in Beijing, China: A four-year retrospective study. Antimicrob. Resist. Infect. Control.

[14] Ayobami O., Willrich N., Reuss A., Eckmanns T., Markwart R. (2020). The ongoing challenge of vancomycin-resistant *Enterococcus faecium* and *Enterococcus faecalis* in Europe: An epidemiological analysis of bloodstream infections. Emerg. Microbes Infect..

[15] Billington E.O., Phang S.H., Gregson D.B., Pitout J.D.D., Ross T., Church D.L., Laupland K.B., Parkins M.D. (2014). Incidence, Risk Factors, and Outcomes for *Enterococcus* spp. Blood Stream Infections: A Population-Based Study. Int. J. Infect. Dis..

[16] Pinholt M., Østergaard C., Arpi M., Bruun N.E., Schønheyder H.C., Gradel K.O., Søgaard M., Knudsen J.D. (2014). Incidence, clinical characteristics and 30-day mortality of enterococcal bacteraemia in Denmark 2006–2009: A population-based cohort study. Clin. Microbiol. Infect..

[17] Zhou X., Willems R.J.L., Friedrich A.W., Rossen J.W.A., Bathoorn E. (2020). *Enterococcus faecium*: From microbiological insights to practical recommendations for infection control and diagnostics. Antimicrob. Resist. Infect. Control.

[18] Foka F.E.T., Mienie C., Bezuidenhout C.C., Ateba C.N. (2020). Complete Genomic Analysis of VRE From a Cattle Feedlot: Focus on 2 Antibiotic Resistance. Front. Microbiol..

[19] Janice J., Wagner T.M., Olsen K., Hegstad J., Hegstad K. (2024). Emergence of vancomycin-resistant *enterococci* from vancomycin-susceptible *enterococci* in hospitalized patients under antimicrobial therapy. J. Glob. Antimicrob. Resist..

[20] Mitchell S.L., Mattei L.M., Alby K. (2017). Whole genome characterization of a naturally occurring vancomycin-dependent *Enterococcus faecium* from a patient with bacteremia. Infect. Genet. Evol..

[21] Buultjens A.H., Lam M.M.C., Ballard S., Monk I.R., Mahony A.A., Grabsch E.A., Grayson M.L., Pang S., Coombs G.W., Robinson J.O. (2017). Evolutionary origins of the emergent ST796 clone of vancomycin resistant *Enterococcus faecium*. PeerJ.

[22] Liu S., Li Y., He Z., Wang Y., Wang J., Jin D. (2022). A molecular study regarding the spread of *vanA* vancomycin-resistant *Enterococcus faecium* in a tertiary hospital in China. J. Glob. Antimicrob. Resist..

[23] Franyó D., Kocsi B., Lesinszki V., Pászti J., Kozák A., Bukta E.E., Szabó J., Dombrádi Z. (2018). Characterization of Clinical Vancomycin-Resistant *Enterococcus faecium* Isolated in Eastern Hungary. Microb. Drug Resist..

[24] Chopjitt P., Boueroy P., Jenjaroenpun P., Wongsurawat T., Hatrongjit R., Kerdsin A., Sunthamala N. (2023). Genomic characterization of vancomycin-resistant *Enterococcus faecium* clonal complex 17 isolated from urine in tertiary hospitals in Northeastern Thailand. Front. Microbiol..

[25] Saengsuwan P., Singkhamanan K., Madla S., Ingviya N., Romyasamit C. (2021). Molecular epidemiology of vancomycinresistant *Enterococcus faecium* clinical isolates in a tertiary care hospital in southern Thailand: A retrospective study. PeerJ.

[26] Kuo A.J., Shu J.C., Liu T.P., Lu J.J., Lee M.H., Wu T.S., Su L.H., Wu T.L. (2018). Vancomycin-resistant *Enterococcus faecium* at a university hospital in Taiwan, 2002–2015: Fluctuation of genetic populations and emergence of a new structure type of the Tn1546-like element. J. Microbiol. Immunol. Infect..

[27] Oh S., Nam S.K., Chang H.E., Park K.U. (2022). Comparative Analysis of Short- and Long-Read Sequencing of Vancomycin-Resistant *Enterococci* for Application to Molecular Epidemiology. Front. Cell. Infect. Microbiol..

[28] Zhou W., Zhou H., Sun Y., Gao S., Zhang Y., Cao X., Zhang Z., Shen H., Zhang C. (2020). Characterization of clinical *enterococci* isolates, focusing on the vancomycin-resistant *enterococci* in a tertiary hospital in China: Based on the data from 2013 to 2018. BMC Infect. Dis..

[29] Şenol F.F., Tanrıverdi E.S., Aytaç Ö., Aşçı Toraman Z., Otlu B. (2023). An Outbreak of Vancomycin-Resistant *Enterococci* in a City Hospital Intensive Care Unit: Molecular Characterization of Resistance. Medicina.

[30] Yang J., Yuan Y., Tang M., Liu L., Yang K., Liu J. (2019). Phenotypic and genetic characteristics of vancomycin-resistant *Enterococcus faecium*. Microb. Pathog..

[31] Tong J., Jiang Y., Xuehang H.X., Li J., Shuaibing Z., Yu Y.W., Qiu Y. (2021). In vitro Antimicrobial Activity of Fosfomycin, Rifampin, Vancomycin, Daptomycin Alone and in Combination Against Vancomycin-Resistant *Enterococci*. Drug Des. Dev. Ther..

[32] Taji A., Heidari H., Ebrahim-Saraie H.S., Sarvari J., Motamedifar M. (2019). High prevalence of vancomycin and high-level gentamicin resistance in *Enterococcus faecalis* isolates. Acta Microbiol. Et Immunol. Hung..

[33] Hemapanpairoa J., Changpradub D., Thunyaharn S., Santimaleeworagun W. (2019). Vancomycin-resistant enterococcal infection in a Thai university hospital: Clinical characteristics, treatment outcomes, and synergistic effect. Infect. Drug Resist..

[34] Cairns K.A., Udy A.A., Peel T.N., Abbott I.J., Dooley M.J., Peleg A.Y. (2023). Therapeutics for Vancomycin-Resistant Enterococcal Bloodstream Infections. Clin. Microbiol. Rev..

[35] Stellfox M.E., Van Tyne D. (2022). Last Bacteria Standing: VREfm Persistence in the Hospitalized Gut. mBio.

[36] Lin P.Y., Chan S.Y., Stern A., Chen P.H., Yang H.C. (2023). Epidemiological profiles and pathogenicity of Vancomycin-resistant *Enterococcus faecium* clinical isolates in Taiwan. PeerJ.

[37] Salamandane A., Cahango G., Muetanene B.A., Malfeito-Ferreira M., Brito L. (2023). Multidrug Resistance in *Enterococci* Isolated from Cheese and Capable of Producing Benzalkonium Chloride-Resistant Biofilms. Biology.

[38] Grudlewska-Buda K., Skowron K., Bauza-Kaszewska J., Budzyńska A., Wiktorczyk-Kapischke N., Wilk M., Wujak M., Paluszak Z. (2023). Assessment of antibiotic resistance and biofilm formation of *Enterococcus* species isolated from different pig farm environments in Poland. BMC Microbiol..

[39] Goudarzi M., Mobarez A.M., Najar-Peerayeh S., Mirzaee M. (2018). Prevalence of biofilm formation and vancomycin-resistant genes among *Enterococcus faecium* isolated from clinical and environmental specimens in Lorestan hospitals. Iran. J. Microbiol..

[40] Akhter J., Ahmed S., Saleh A.A., Anwar S. (2014). Antimicrobial resistance and in vitro biofilm-forming ability of *Enterococci* spp. isolated from urinary tract infection in a tertiary care hospital in Dhaka. Bangladesh Med. Res. Counc. Bull..

[41] Hashem Y.A., Amin H.M., Essam T.M., Yassin A.S., Aziz R.K. (2017). Biofilm formation in *enterococci*: Genotype-phenotype correlations and inhibition by vancomycin. Sci. Rep..

[42] Wei M., Wang P., Li T., Wang Q., Su M., Gu L., Wang S. (2023). Antimicrobial and antibiofilm effects of essential fatty acids against clinically isolated vancomycin-resistant *Enterococcus faecium*. Front. Cell. Infect. Microbiol..

[43] Arndt F., Siems K., Walker S.V., Bryan N.C., Leuko S., Moeller R., Boschert A.L. (2024). Systematic screening of 42 vancomycin-resistant *Enterococcus faecium* strains for resistance, biofilm, and desiccation in simulated microgravity. Npj Microgravity.

[44] Kim S.H., Cho S.Y., Kim H.M., Huh K., Kang C.I., Peck K.R., Chung D.R. (2021). Sequence type 17 is a predictor of subsequent bacteremia in vancomycin-resistant *Enterococcus faecium*-colonized patients: A retrospective cohort study. Antimicrob. Resist. Infect. Control.

[45] Abdelbary M.H.H., Senn L., Greub G., Chaillou G., Moulin E., Blanc D.S. (2019). Whole-genome sequencing revealed independent emergence of vancomycin-resistant *Enterococcus faecium* causing sequential outbreaks over 3 years in a tertiary care hospital. Eur. J. Clin. Microbiol. Infect. Dis..

[46] Caplunik-Pratsch A., Kieninger B., Donauer V.A., Brauer J.M., Meier V.M.K., Seisenberger C., Rath A., Loibl D., Eichner A., Fritsch J. (2024). Introduction and spread of vancomycin-resistant *Enterococcus faecium* (VREfm) at a German tertiary care medical center from 2004 until 2010: A retrospective whole-genome sequencing (WGS) study of the molecular epidemiology of VREfm. Antimicrob. Resist. Infect. Control.

[47] Jozefíková A., Valček A., Šoltys K., Nováková E., Bujdáková H. (2022). Persistence and multi-ward dissemination of vancomycin-resistant *Enterococcus faecium* ST17 clone in hospital settings in Slovakia 2017–2020. Int. J. Antimicrob. Agents.

[48] Yashar J., Igor S., Monika J., Jan K., Adriana L., Andrea K., Juraj P. (2022). First report of nosocomial outbreak of vancomycin-resistant Enterococcus faecium infection among COVID-19 patients hospitalized in a non-intensive care unit ward in Slovakia. Bratislava Medical Journal.

[49] Xanthopoulou K., Peter S., Tobys D., Behnke M., Dinkelacker A.G., Eisenbeis S., Falgenhauer J., Falgenhauer L., Fritzenwanker M., Gölz H. (2020). Vancomycin-resistant *Enterococcus faecium* colonizing patients on hospital admission in Germany: Prevalence and molecular epidemiology. J. Antimicrob. Chemother..

[50] Lisotto P., Couto N., Rosema S., Lokate M., Zhou X., Bathoorn E., Harmsen H.J.M., Friedrich A.W., Rossen J.W.A., Chlebowicz-Fliss M.A. (2021). Molecular Characterisation of Vancomycin-Resistant *Enterococcus faecium* Isolates Belonging to the Lineage ST117/CT24 Causing Hospital Outbreaks. Front. Microbiol..

[51] Rios R., Reyes J., Carvajal L.P., Rincon S., Panesso D., Echeverri A.M., Dinh A., Kolokotronis S.O., Narechania A., Tran T.T. (2020). Genomic Epidemiology of Vancomycin-Resistant *Enterococcus faecium* (VRE*fm*) in Latin America: Revisiting The Global VRE Population Structure. Sci. Rep..

[52] Leong K.W.C., Kalukottege R., Cooley L.A., Anderson T.L., Wells A., Langford E., O’Toole R.F. (2020). State-Wide Genomic and Epidemiological Analyses of Vancomycin-Resistant *Enterococcus faecium* in Tasmania’s Public Hospitals. Front. Microbiol..

[53] Akpaka P.E., Kissoon S., Jayaratne P., Wilson C., Golding G.R., Nicholson A.M., Lewis D.B., Hermelijn S.M., Wilson-Pearson A., Smith A. (2017). Genetic characteristics and molecular epidemiology of vancomycin-resistant *Enterococci* isolates from Caribbean countries. PLoS ONE.

[54] Pham L.T.T., Pharkjaksu S., Chongtrakool P., Suwannakarn K., Ngamskulrungroj P. (2019). A predominance of clade 17 Candida albicans isolated from hemocultures in a tertiary care hospital in Thailand. Front. Microbiol..

[55] Choi D.G., Baek J.H., Han D.M., Khan S.A., Jeon C.O. (2024). Comparative pangenome analysis of *Enterococcus faecium* and *Enterococcus* lactis provides new insights into the adaptive evolution by horizontal gene acquisitions. BMC Genom..

[56] Rahman M.H., El Zowalaty M.E., Falgenhauer L., Khan M.F.R., Alam J., Popy N.N., Rahman M.B. (2024). Draft genome sequences of clinical mastitis-associated *Enterococcus faecalis* and *Enterococcus faecium* carrying multiple antimicrobial resistance genes isolated from dairy cows. J. Glob. Antimicrob. Resist..

[57] Hashimoto Y., Kurushima J., Nomura T., Tanimoto K., Tamai K., Yanagisawa H., Shirabe K., Ike Y., Tomita H. (2018). Dissemination and genetic analysis of the stealthy *vanB* gene clusters of *Enterococcus faecium* clinical isolates in Japan. BMC Microbiol..

[58] Islam M., Sharon B., Abaragu A., Sistu H., Akins R.L., Palmer K. (2023). Vancomycin Resistance in *Enterococcus faecium* from the Dallas, Texas, Area Is Conferred Predominantly on pRUM-Like Plasmids. mSphere.

[59] Shen J., Long X., Jiang Q., Xu H., Wei Q., Shi Q., Liu Y., Xu S., Ma X., Li L. (2023). Genomic Characterization of a Vancomycin-Resistant Strain of *Enterococcus faecium* Harboring a *rep2* Plasmid. Infect. Drug Resist..

[60] Merlo T.P., Dabul A.N.G., Camargo I.L.B.C. (2015). Different VanA Elements in *E. faecalis* and in *E. faecium* Suggest at Least Two Origins of Tn1546 Among VRE in a Brazilian Hospital. Microb. Drug Resist..

[61] Sun L., Xu J., Wang W., He F. (2020). Emergence of *vanA*-Type Vancomycin-Resistant *Enterococcus faecium* ST 78 Strain with a *rep2*-Type Plasmid Carrying a Tn1546-Like Element Isolated from a Urinary Tract Infection in China. Infect. Drug Resist..

[62] Arredondo-Alonso S., Top J., Corander J., Willems R.J.L., Schürch A.C. (2021). Mode and dynamics of *vanA*-type vancomycin resistance dissemination in Dutch hospitals. Genome Med..

[63] Fujiya Y., Harada T., Sugawara Y., Akeda Y., Yasuda M., Masumi A., Hayashi J., Tanimura N., Tsujimoto Y., Shibata W. (2021). Transmission dynamics of a linear *vanA*-plasmid during a nosocomial multiclonal outbreak of vancomycin-resistant *enterococci* in a non-endemic area, Japan. Sci. Rep..

[64] Egan S.A., Kavanagh N.L., Shore A.C., Mollerup S., Samaniego Castruita J.A., O’Connell B., McManus B.A., Brennan G.I., Pinholt M., Westh H. (2022). Genomic analysis of 600 vancomycin-resistant *Enterococcus faecium* reveals a high prevalence of ST80 and spread of similar *vanA* regions via IS1216E and plasmid transfer in diverse genetic lineages in Ireland. J. Antimicrob. Chemother..

[65] Saenhom N., Boueroy P., Chopjitt P., Hatrongjit R., Kerdsin A. (2022). Distinguishing Clinical *Enterococcus faecium* Strains and Resistance to Vancomycin Using a Simple In-House Screening Test. Antibiotics.

[66] Jackson C.R., Fedorka-Cray P.J., Barrett J.B. (2004). Use of a genus- and species-specific multiplex PCR for identification of *enterococci*. J. Clin. Microbiol..

[67] CLSI Clinical and Laboratory Standards Institute (CLSI) (2024). Performance Standards for Antimicrobial Susceptibility Testing.

[68] EUCAST The European Committee on Antimicrobial Susceptibility Testing (2024). Breakpoint Tables for Interpretation of MICs and Zone Diameters.

[69] Pérez-Hernández X., Méndez-Álvarez S., Claverie-Martín F. (2002). A PCR assay for rapid detection of vancomycin-resistant *enterococci*. Diagn. Microbiol. Infect. Dis..

[70] Chopjitt P., Tangthong P., Kongkaem J., Wonkyai P., Charoenwattanamaneechai A., Khankhum S., Sunthamala P., Kerdsin A., Sunthamala N. (2025). Molecular characterization and genotype of multi-drug resistant Staphylococcus epidermidis in nasal carriage of young population, Mahasarakham, Thailand. Biomol. Biomed..

[71] Homan W.L., Tribe D., Poznanski S., Li M., Hogg G., Spalburg E., Van Embden J.D.A., Willems R.J.L. (2002). Multilocus sequence typing scheme for *Enterococcus faecium*. J. Clin. Microbiol..

[72] Wick R.R., Judd L.M., Holt K.E. (2019). Performance of neural network basecalling tools for Oxford Nanopore sequencing. Genome Biol..

[73] De Coster W., Rademakers R. (2023). NanoPack2: Population-scale evaluation of long-read sequencing data. Bioinformatics.

[74] Lang J., Zhu R., Sun X., Zhu S., Li T., Shi X., Sun Y., Yang Z., Wang W., Bing P. (2021). Evaluation of the MGISEQ-2000 Sequencing Platform for Illumina Target Capture Sequencing Libraries. Front. Genet..

[75] Gurevich A., Saveliev V., Vyahhi N., Tesler G. (2013). QUAST: Quality assessment tool for genome assemblies. Bioinformatics.

[76] Tanizawa Y., Fujisawa T., Nakamura Y. (2018). DFAST: A flexible prokaryotic genome annotation pipeline for faster genome publication. Bioinformatics.

[77] Larsen M.V., Cosentino S., Rasmussen S., Friis C., Hasman H., Marvig R.L., Jelsbak L., Sicheritz-Pontén T., Ussery D.W., Aarestrup F.M. (2012). Multilocus sequence typing of total-genome-sequenced bacteria. J. Clin. Microbiol..

[78] Alcock B.P., Huynh W., Chalil R., Smith K.W., Raphenya A.R., Wlodarski M.A., Edalatmand A., Petkau A., Syed S.A., Tsang K.K. (2023). CARD 2023: Expanded curation, support for machine learning, and resistome prediction at the Comprehensive Antibiotic Resistance Database. Nucleic Acids Res..

[79] Bortolaia V., Kaas R.S., Ruppe E., Roberts M.C., Schwarz S., Cattoir V., Philippon A., Allesoe R.L., Rebelo A.R., Florensa A.F. (2020). ResFinder 4.0 for predictions of phenotypes from genotypes. J. Antimicrob. Chemother..

[80] Carattoli A., Hasman H. (2020). PlasmidFinder and In Silico pMLST: Identification and Typing of Plasmid Replicons in Whole-Genome Sequencing (WGS). Methods Mol. Biol..

[81] Camacho C., Coulouris G., Avagyan V., Ma N., Papadopoulos J., Bealer K., Madden T.L. (2009). BLAST+: Architecture and applications. BMC Bioinform..

[82] Clausen P.T.L.C., Aarestrup F.M., Lund O. (2018). Rapid and precise alignment of raw reads against redundant databases with KMA. BMC Bioinform..

[83] Jolley K.A., Maiden M.C.J. (2010). BIGSdb: Scalable analysis of bacterial genome variation at the population level. BMC Bioinform..

[84] Zhou Z., Alikhan N.F., Sergeant M.J., Luhmann N., Vaz C., Francisco A.P., Carriço J.A., Achtman M. (2018). GrapeTree: Visualization of core genomic relationships among 100,000 bacterial pathogens. Genome Res..

[85] (2023). Torsten Seemann Snippy [Perl]. https://github.com/tseemann/snippy.

[86] Letunic I., Bork P. (2021). Interactive Tree of Life (iTOL) v5: An online tool for phylogenetic tree display and annotation. Nucleic Acids Res..

